# Supplementary material for: Unveiling charge utilization mechanisms in ferroelectric for water splitting
Source: Nat Commun. 2025 Feb 11;16:1515. doi: 10.1038/s41467-025-56359-y (PMC11810992; doi:10.1038/s41467-025-56359-y)
Supplement: Supplementary file 1 — Supplementary Information [file 41467_2025_56359_MOESM1_ESM.pdf]

Supplementary Information

Unveiling Charge Utilization Mechanisms in Ferroelectric for Water Splitting

Jie Zhang[1,2], Yong Liu[1], Thomas Dittrich[3], Zhuan Wang[4], Pengxiang Ji[2,7], Mingrun Li[1], Na Ta[1], Hongyan Zhang[5], Chao Zhen[6], Yanjun Xu[4], Dongfeng Li[1,2], Zhendong Feng[1,2] Zheng Li[1], Yaling Luo[1,2], Junhao Cui[1,2], Dong Su[7], Yuxiang Weng[4], Gang Liu[6], Xiuli Wang[1], Fengtao Fan[1]*, Can Li[1,2]*

[1]State Key Laboratory of Catalysis, Dalian National Laboratory for Clean Energy, The Collaborative Innovation Centre of Chemistry for Energy Materials (iChEM), Dalian Institute of Chemical Physics, Chinese Academy of Sciences, Dalian, 116023, China.

[2]University of Chinese Academy of Sciences, Beijing, 100049, China.

[3]Helmholtz-Zentrum Berlin für Materialien und Energie GmbH, Institut für Silizium-Photovoltaik, Kekuléstr. 5, Berlin, 12489, Germany.

[4]Laboratory of Soft Matter Physics, Beijing National Laboratory for Condensed Matter Physics, Institute of Physics, Chinese Academy of Sciences, Beijing, 100190, China.

[5]Institutional Center for Shared Technologies and Facilities, Dalian Institute of Chemical Physics, Chinese Academy of Sciences, Dalian, 116023, China.

[6]Shenyang National Laboratory for Materials Science, Institute of Metal Research, Chinese Academy of Sciences, 72 Wenhua Road, Shenyang, 110016, China.

[7]Beijing National Laboratory for Condensed MatterPhysics, Institute of Physics, Chinese Academy of Sciences, Beijing, 100190, China.

Corresponding Authors

Fengtao Fan

Email: ftfan@dicp.ac.cn

Can Li

Email: canli@dicp.ac.cn

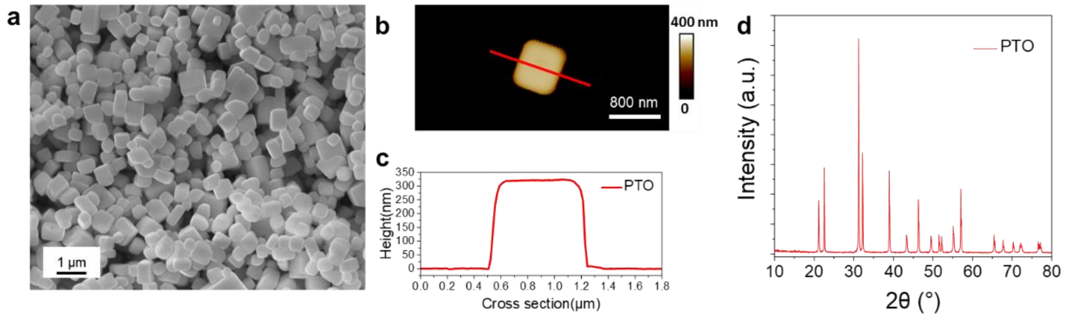

Fig. S1 Morphology and the structure of PTO. **a**, SEM image of PTO powders. **b**, AFM image of a single particle. **c**, AFM cross-section data of the cutline in (**b**). **d**, XRD pattern of PTO powders.

SEM and AFM revealed that PTO particles exhibit a uniform morphology with an approximate size of 600 nm. XRD indicates that PTO has a consistent structural configuration, aligning with the reports in literature (JCPDS 70-0746). The synthesized PTO is in a ferroelectric tetragonal phase with lattice parameters a = b = 0.3905 nm, c = 0.4156 nm.

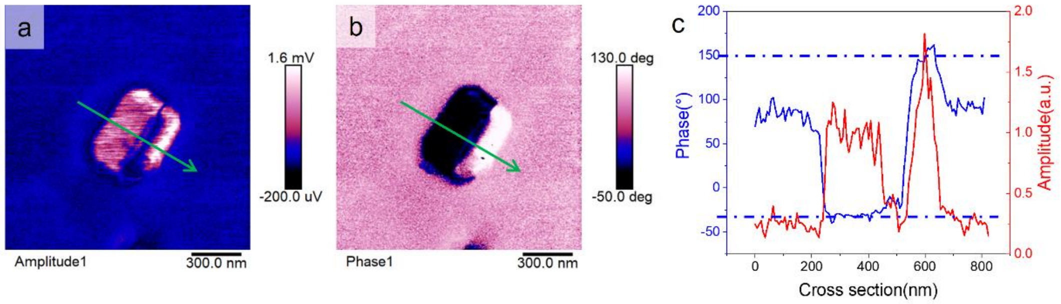

Fig. S2 PFM data of PTO after annealing at 950 °C. **a**, PFM amplitude of PTO particle after annealing at 950 °C. **b**, PFM phase of PTO particle after annealed at 950 °C. **c**, amplitude (red line) and phase (blue libn) of PTO particle align with the green arrow in (**a**) and (**b**).

The Curie temperature of PTO is approximately 495 °C. During annealing at 950 °C, PTO transitioned from tetragonal to cubic phase. Upon subsequent cooling below the Curie temperature, PTO reverted to the ferroelectric tetragonal phase. The observed 180 °C phase difference indicated the opposite polarization direction in different regions of PTO. Compared with the data in Fig. 1, PTO transitioned from a monodomain to a multidomain structure.

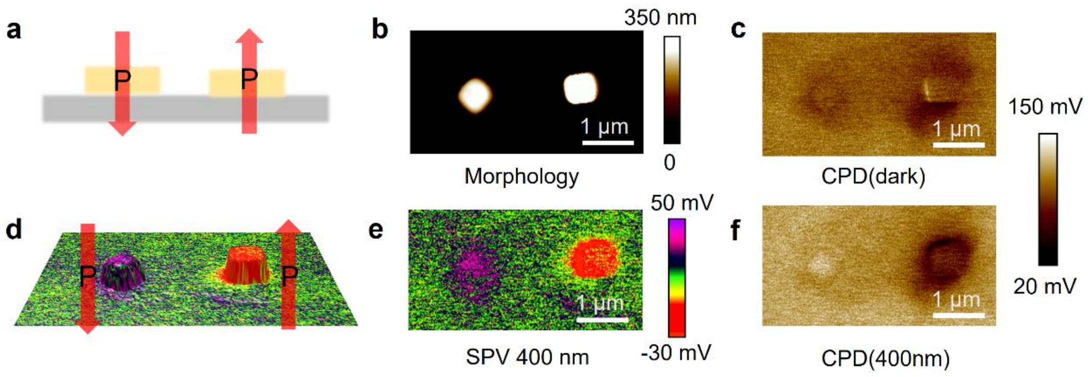

Fig. S3 KPFM images of PTO with different polarization directions. **a**, schematic of the particles tested by KPFM; the red arrows represent the polarization direction. **b**, Morphology of the particles with different polarization directions. **c**, KPFM images of PTO on ITO substrate in the dark; **f**, KPFM images of PTO on ITO substrate under 400 nm light illumination. **d**,**e**, SPV images of the particles. The red color represents electrons transferred to the surface while the magenta color represents holes transferred to the surface.

Fig. S3 presents the KPFM data of PTO samples. Consistent with our earlier findings, the particles on the left exhibit a polarization direction oriented towards the substrate, evidenced by the brighter CPD representative of the separated holes on the surface. Conversely, the particles on the right display the opposite polarization direction, with a darker CPD representative of the separated electrons on the surface. This observation suggests that ferroelectric PTO could selectively separate electrons and holes in distinct directions within a single particle.

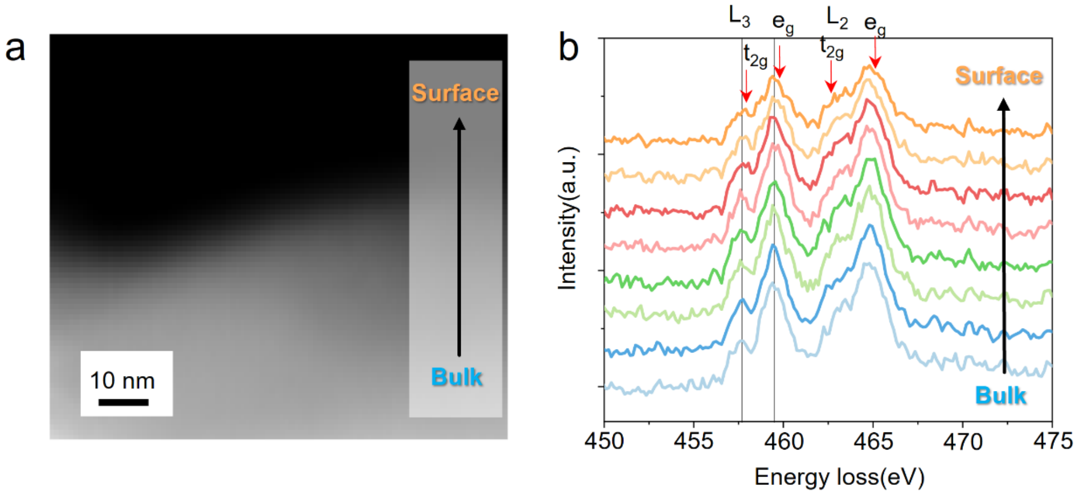

Fig. S4 Surface structure evolution in positive polarization of PTO. **a,** STEM data of PTO surface. **b,** EELS evolution from bulk to surface of PTO.

EELS peaks at 457.6 eV, 459.4 eV, 463.45 eV and 464.8 eV in the bulk PTO represented t_{2g} (L_3) e_g (L_3) t_{2g} (L_2) and e_g (L_2) energy levels, respectively. Moving from the bulk to the surface, the splitting of t_{2g} and e_g in L_3 energy level was decreased. These reduced splitting were associated with the decreased degeneracy of Ti d orbits within oxygen octahedron field, which in turn is related to the surface distortion of the unit cell. These distortions are likely caused by defects in the surface region.

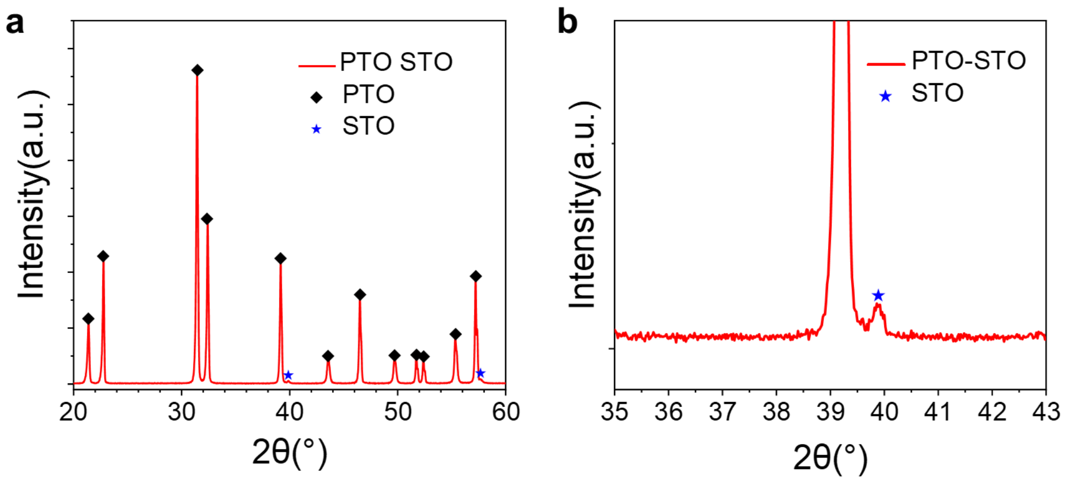

Fig. S5 Structure of PTO-STO samples. **a**, XRD of PTO-STO. The peaks marked with black rhombuses are the main peaks of PTO, and the small peaks marked with blue stars are the distinguishable peaks of STO to identify it from PTO. **b**, the magnified data of a) in 2θ range between 35-43°.

Fig. S5 presents XRD patterns of PTO-STO samples. STO exhibits a cubic structure (JCPDS 79-0176) with unit cell parameters of a = b = c = 0.39046 nm closely resembling those of PTO (a = b = 0.3905 nm, c = 0.4156). Consequently, most of the peaks of STO overlap with those of PTO except the (111) facet peak at 39.96° marked with blue star is discernible.

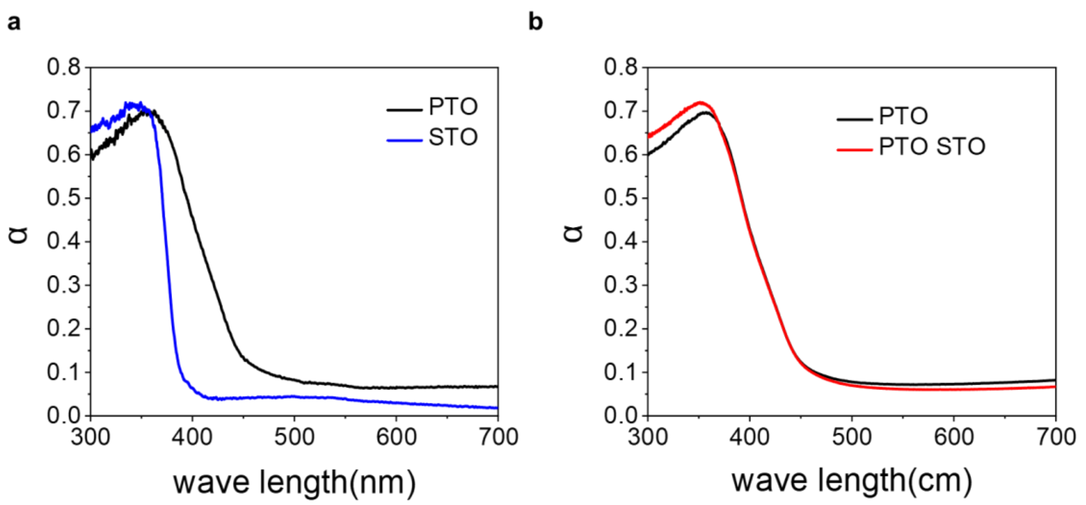

Fig. S6 Diffuse reflectance UV-vis absorption spectra s of the different samples. **a**, bare PTO and STO samples. **b**, PTO before and after growing STO film.

As shown in Fig. S6a, PTO exhibits a broader absorption range than STO, while STO shows a higher absorption coefficient in the 300-350 nm region. After growth of STO on PTO, the absorption spectrum shows cumulative effect of PTO and STO.

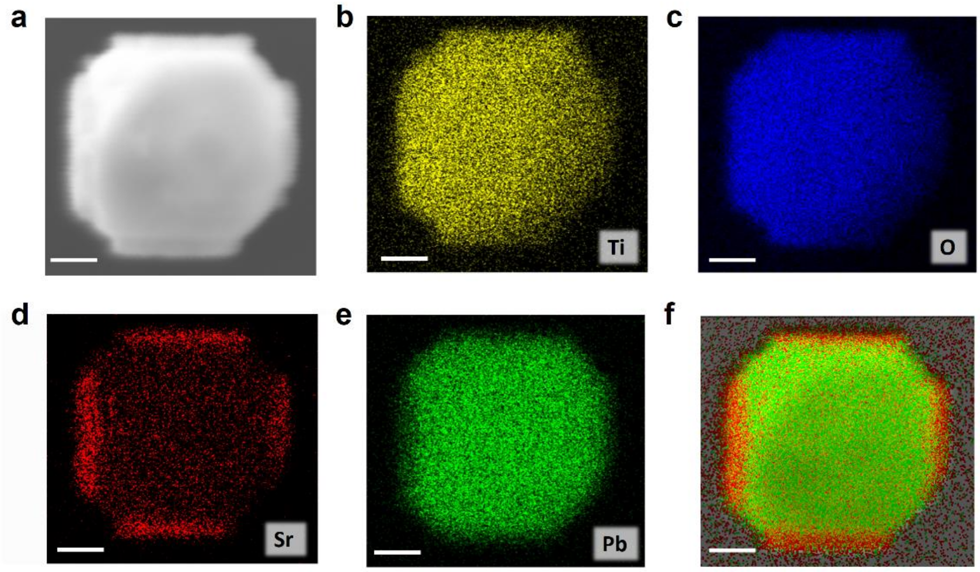

Fig. S7 EDS data of selective growth of STO on PTO. **a**, SEM image of PTO-STO. Images of the elements distribution are shown in (**b**) to (**e**): **b**, Ti, **c**, O, **d**, Sr, **e**, Pb, **f**, combination of Pb and Sr. (The scale bar was 100 nm)

Fig. S7 provides image evidences for spatial location of PTO and STO. It can be seen that Ti and O exhibit uniform distribution, Sr is predominantly distributed on the outer side, and Pb is concentrated in the inner region of the particle. As a result, STO is grown on the surface of PTO. Further corroborating information can be seen in SEM images shown in Fig. 3a, and Fig. 3b, which collectively demonstrate that STO covers both the positively polarized facets and the nonpolarized facets of PTO, leaving negative facet exposed.

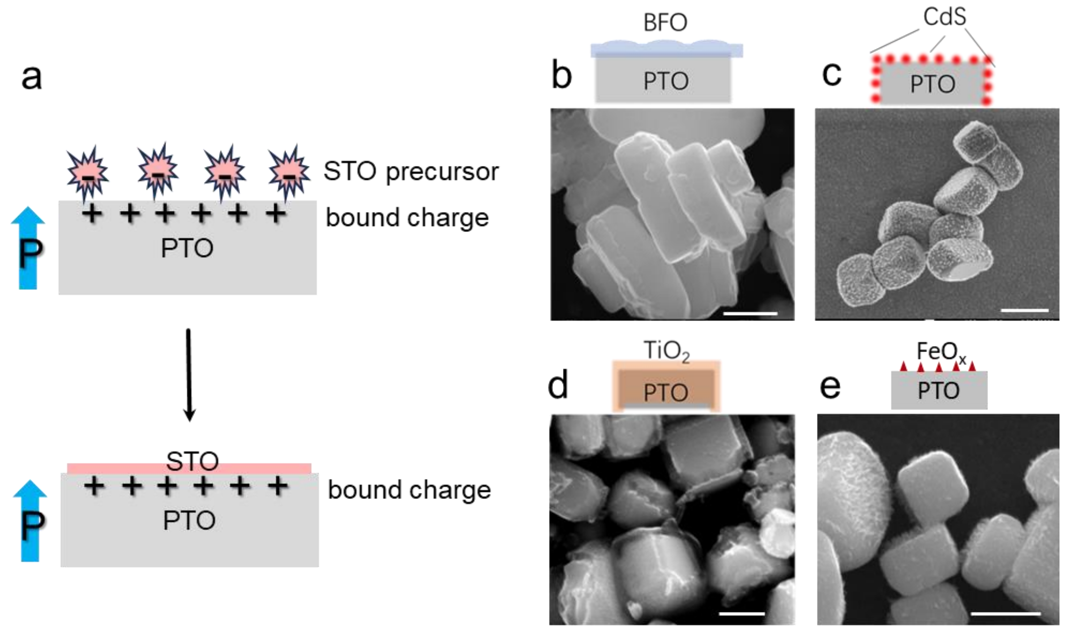

Fig. S8 Growth mechanism of STO on PTO. **a**, schematic of STO growing mechanism on PTO surface. **b**, $BiFeO_3$(BFO), **c**, CdS **d**, TiO_2, **e**, FeO_x selectively growth on PTO. (The scales of the bar: (**b**) 500 nm, (**c**) 500 nm, (**d**) 500 nm and (**e**) 500 nm)

The growth of STO on PTO was induced by the electrostatic adsorption of STO precursors onto the PTO surface with positive bound charges. As shown in Fig. S8, in the initial stage, negatively charged STO precursors were electrostatic adsorbed onto the positively polarized PTO facet, which contained positive bound charges. Following adsorption, the STO precursors grew into nanolayer films. This electrostatic adsorption mechanism was universal, as evidenced by the selective growth of BFO, CdS, TiO_2 and FeO_x as demonstrated in Fig. S8 b-e.

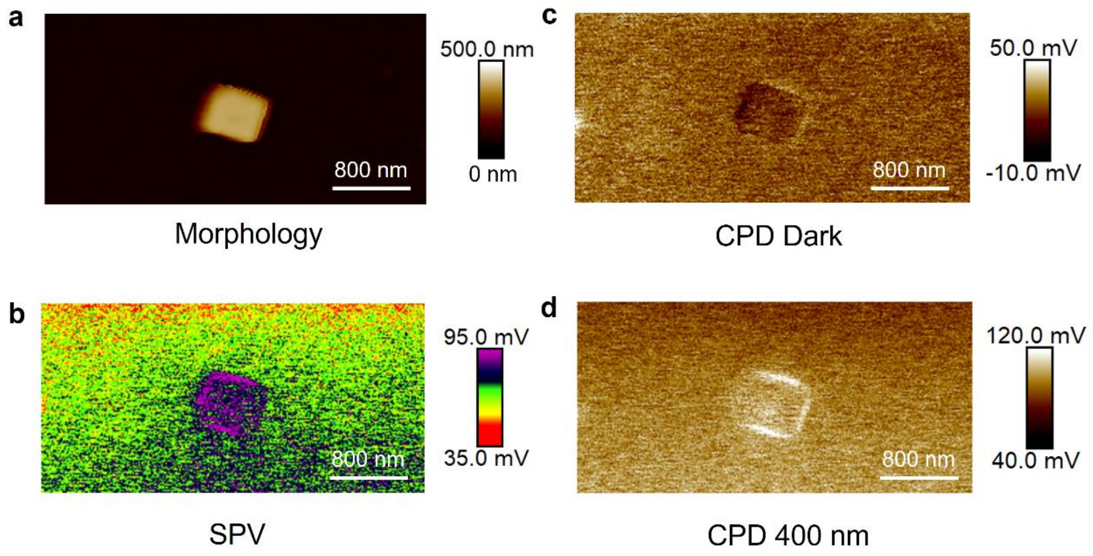

Fig. S9 AFM and SPVM data of PTO-STO samples. **a**, morphology of PTO-STO samples, **c**, and **d**, CPD of PTO-STO particle under dark and 400 nm light illumination, respectively. **b**, SPVM of PTO-STO samples.

Fig. S9 shows the AFM and SPVM images of PTO-STO samples. Notably, there is no discernible STO layer covering the particles based on the AFM morphology characterization. The CPD became brighter under illumination with 400 nm light compared to its dark state (Fig. S9c and Fig. S9d), indicating the presence of a positive SPV signal (Fig. S9b). These results suggest that holes are readily transferred to the STO surface under illumination, a phenomenon consistent with the result of the photodeposition of the MnO_x as shown in Fig. 3e.

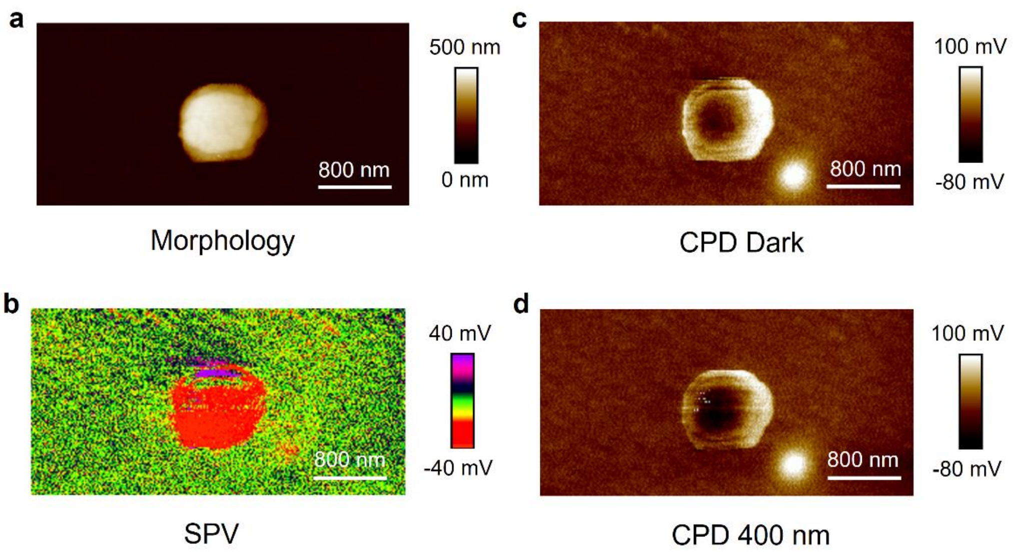

Fig. S10 AFM and SPVM images of PTO-STO samples. **a**, morphology of PTO-STO samples, **c**,**d**, CPD of PTO-STO particle under dark and 400 nm light illumination, respectively. **b**, SPVM of PTO-STO samples.

Fig. S10 shows the AFM and SPVM images of PTO-STO samples. The AFM demonstrates the presence of STO film covering the particles. Notably, The CPD image of the particle became darker under illumination with 400 nm light compared to its dark state (Fig. S10c and Fig. S10d), which is consistent with the negative SPV signal observed (Fig. S10b). These results suggest that photogenerated electrons tend to transfer to the STO surface under illumination, which is consistent with the result of the photodeposition of Ag shown in Fig. 3d.

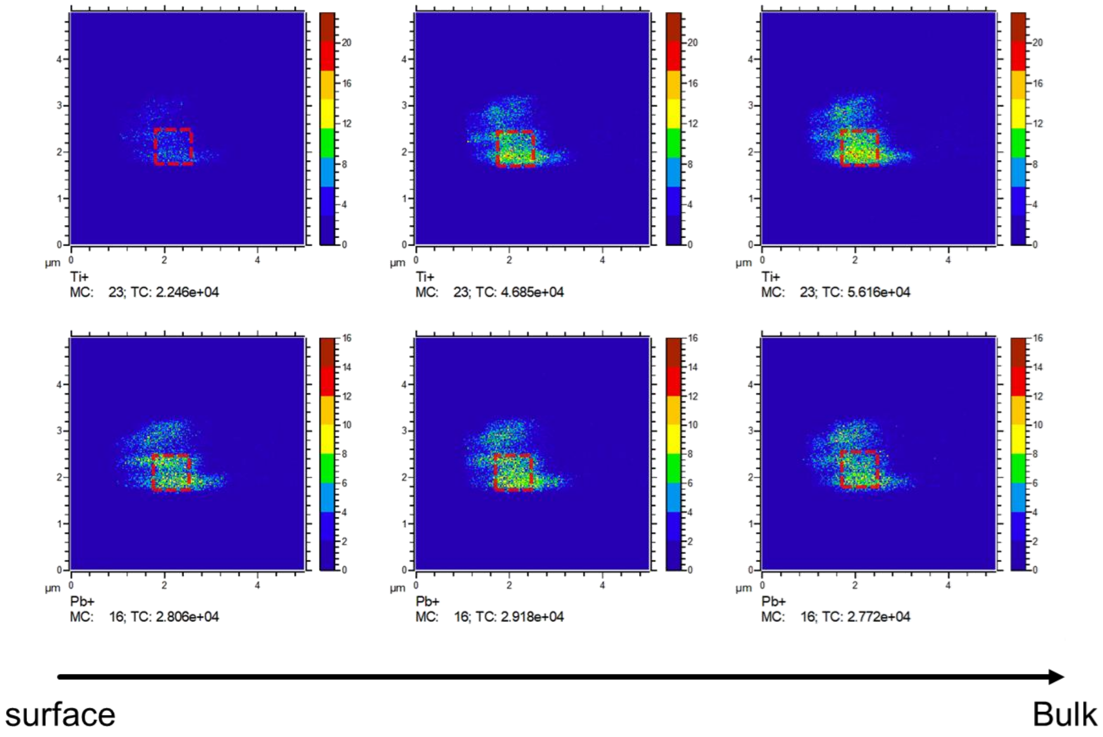

Fig. S11 Element distribution from PTO surface to the bulk. The pictures in the first line and second line show the distribution of Ti and Pb, respectively.

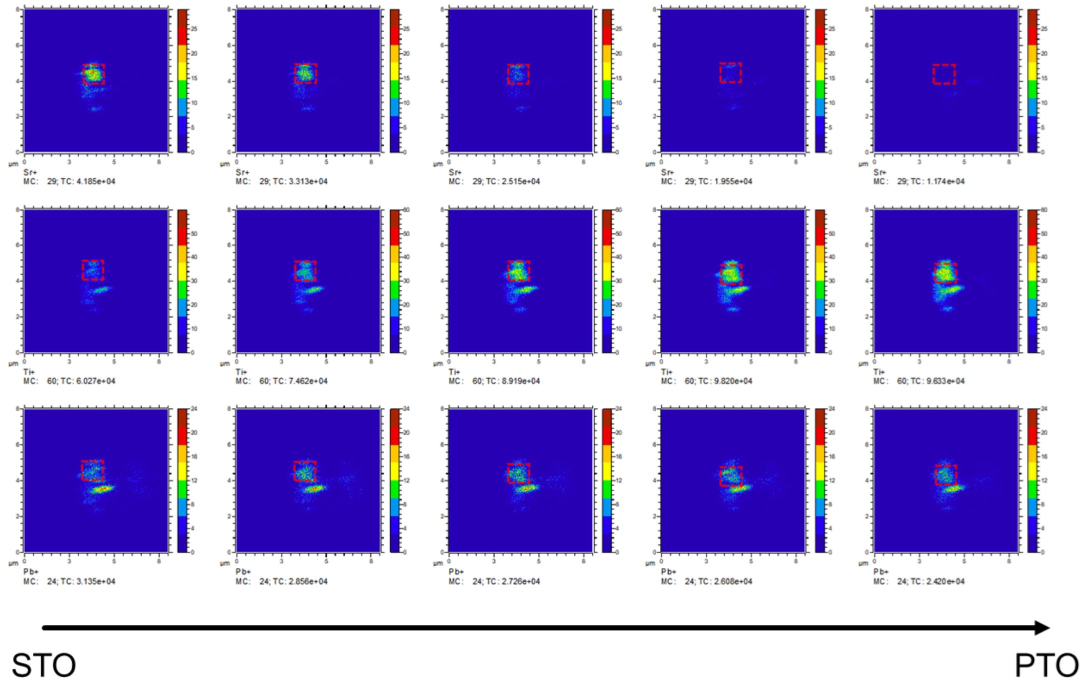

Fig. S12 Element distribution from STO to the bulk of PTO. The pictures in first line show the distribution of Sr. The pictures in second line show the distribution of Ti. The pictures in third line show the distribution of Pb.

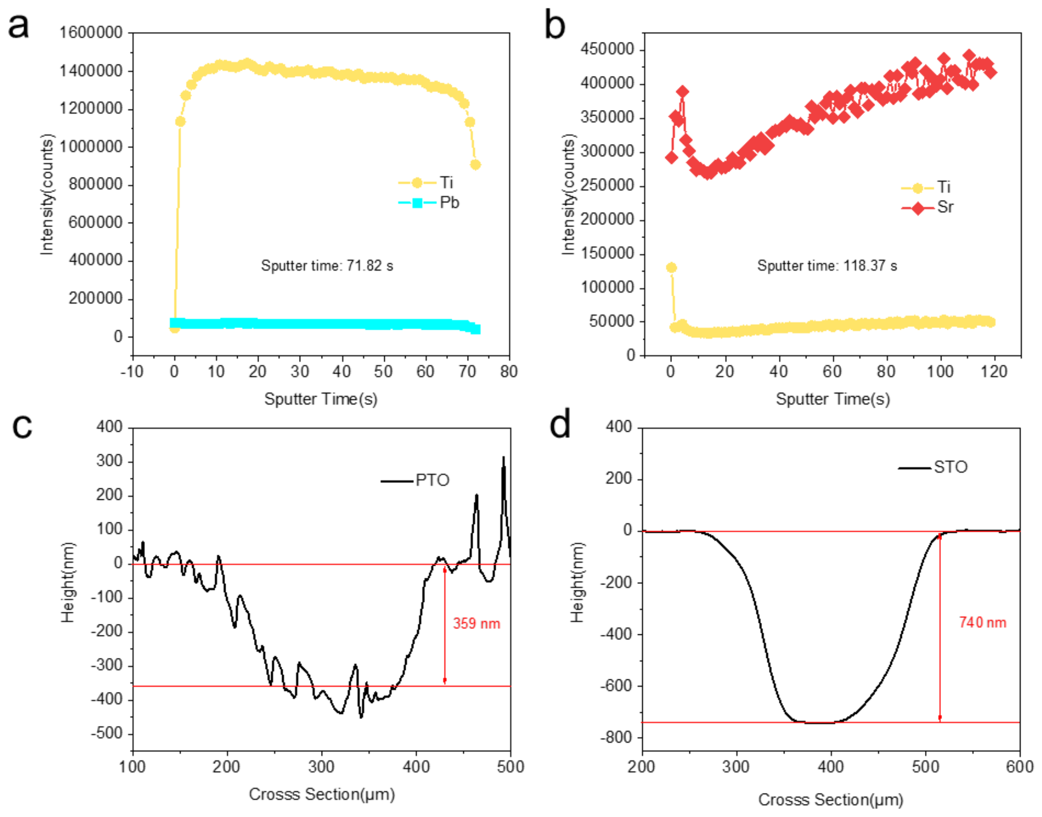

Fig. S13 Calibration of the depth of PTO and STO during TFO-SIMS tests. **a,b,** element evolution of PTO single film and STO single substrate with sputter time, respectively. **c,d,** depth of sputter region after sputter.

Based on the test data, the sputter rates for PTO and STO were determined to be 5 nm/s and 6.25 nm/s, respectively.

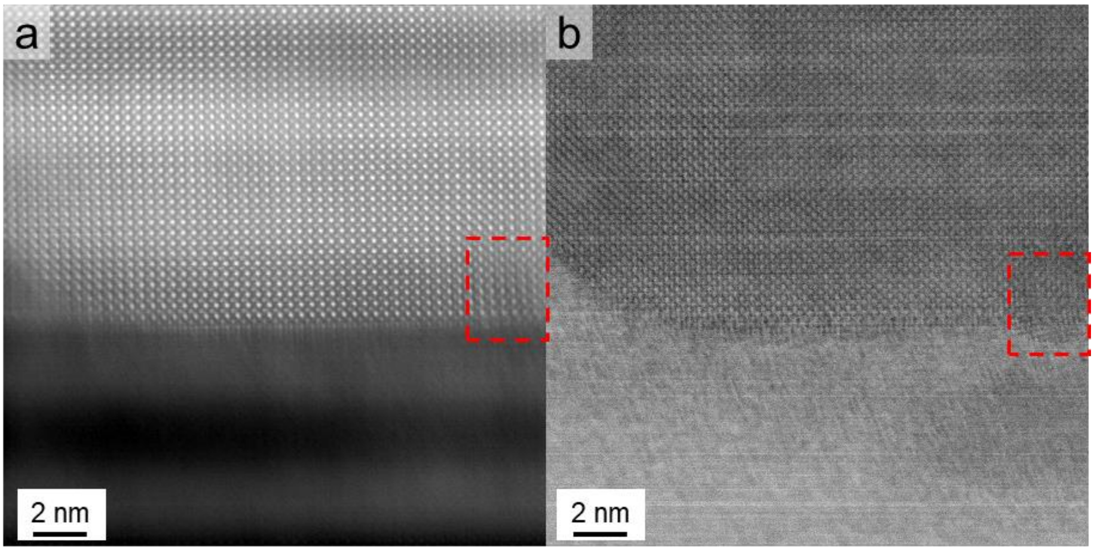

Fig. S14 Surface structure of STO growth on PTO. **a,b,** HAADF-STEM and ABF-STEM data of STO surface.

From STEM, most of the surface regions of STO were well structured. Only the small region showed distortion in surface region, which could be caused by the surface defects.

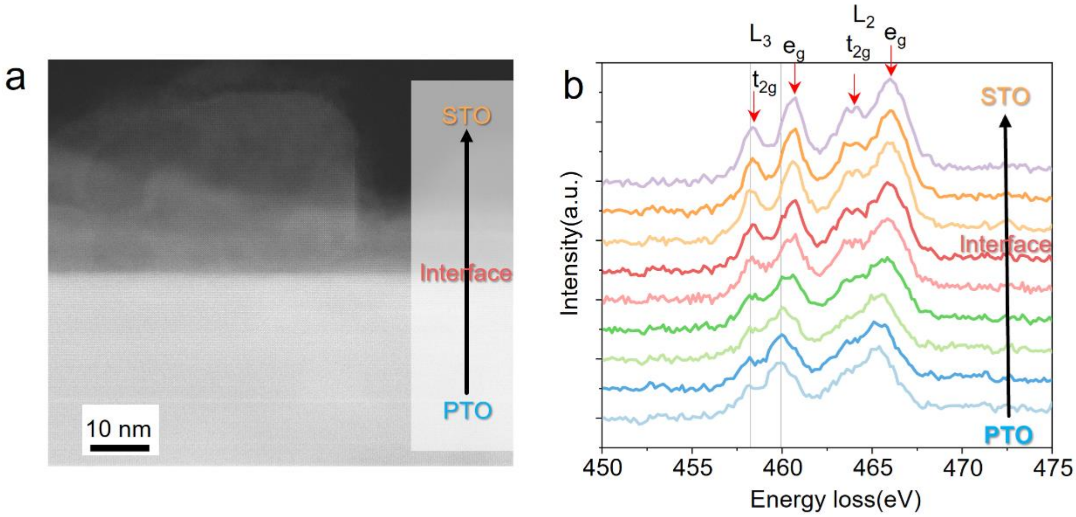

Fig. S15 Surface structure evolution in the interface of PTO. **a,** STEM data of PTO-STO interface. **b,** EELS evolution from PTO to STO.

The structure evolution of PTO was illustrated in Fig. S10b. The splitting of the t_{2g} and e_g peaks splitting of Ti increased from PTO to STO, indicating a reduction in Ti defects and a transition from tetragonal PTO to cubic STO. Notably, unlike the decreased splitting observed at the surface, a significant increase in splitting was observed at the PTO interface at the PTO interface. This suggested that the PTO interface exhibited some cubic properties of STO, as further corroborated by Fig. S16.

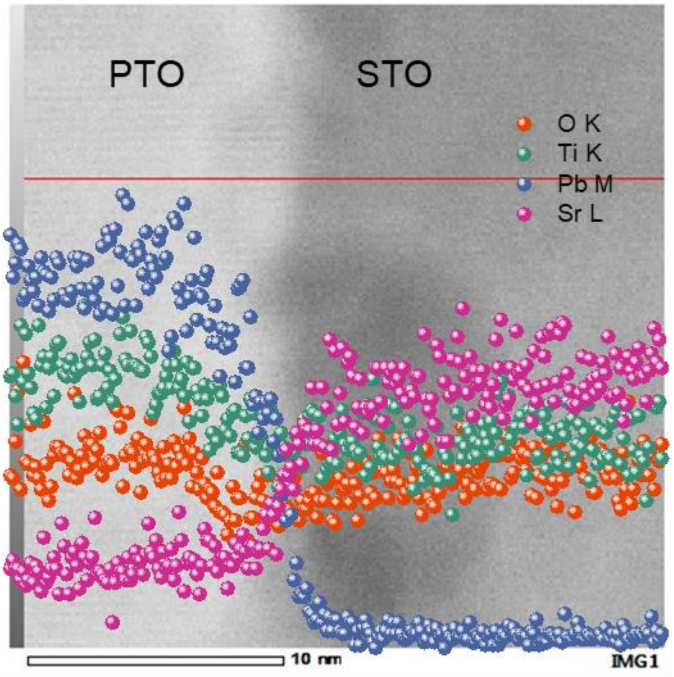

Fig. S16 EDS results near PTO-STO surface. The bar in the SEM was 10 nm.

From EDS data across the interface, the distribution of elements of PTO after modification by STO could be seen. The data reveal that some Pb in the PTO interface was replaced by Sr during the growth of STO. This replacement helped explain the cubic properties observed at the PTO interface, as shown in the EELS data in Fig. S10. It is important to note that the apparent increase in Ti intensity from the PTO interface to the bulk seemingly conflicts with the TOF-SIMS data. This discrepancy arises because the EDS measurements, conducted using STEM, likely captured increased signals due to non-uniform sample thickness caused by the slicing process. The samples were prepared with a diamond blade mounted

on a rotating arm, resulting in arc-shaped sections with varying thicknesses, thus affecting the quantitative data accuracy.

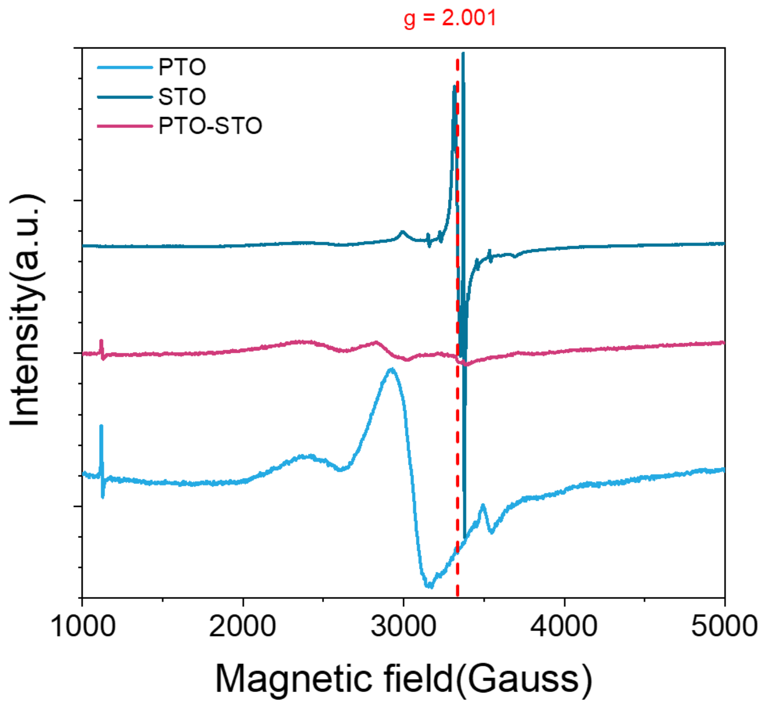

Fig. S17 Defects elimination of PTO after growing STO nanolayers.

After growing STO nanolayers, electron paramagnetic resonance spectroscopy (EPR) peaks dramatically decreased, indicating a decrease in the concentration of defects. In particular, the defect peaks in the PTO-STO samples were only slightly larger than the fluctuation of the EPR baseline, further confirming the presence of fewer defects.

It is worth noting that the PTO samples prepared in this work were subjected to higher temperatures than those in similar studies.[1] Typically, higher temperatures result in increased formation of defects in our PTO samples. However, contrary to this expectation, we found that the defects significantly reduced after the growth of STO nanolayers. We hypothesize that this reduction in defects may be related to the different concentrations of NaOH used during the preparation process. Specifically, the NaOH

concentration we used here was 2 mol/L, which is half of that used in a recent report. This difference in concentration could have contributed to the observed reduction in defects.

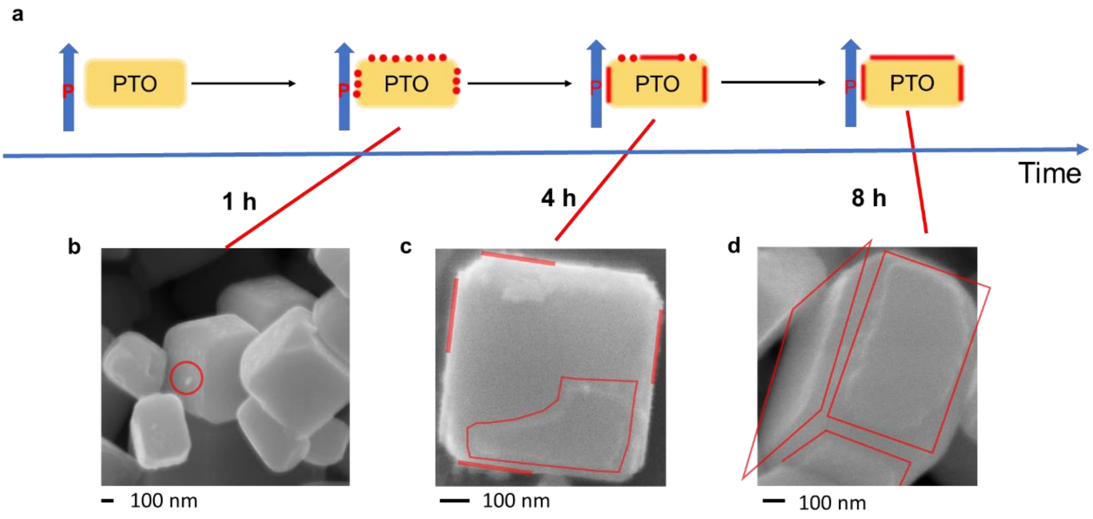

Fig. S18 SEM images of PTO-STO hydrothermally synthesized at different reaction time intervals (first stage:1-8 hours). **a**, schematics of STO growing process on PTO surface, The red regions represent STO. **b**, 1 hour, **c**, 4 hours, **d**, 8 hours. The film in red closed curve is STO. The red lines link the schematics and the SEM data at different stages.

Fig. S18 illustrates the sequential stages in the formation of a STO on PTO surface. Initially, adsorption of the STO precursor on the PTO facet was observed within the first hour. Subsequently, the formation of the film occurred on both nonpolar facet and positive polarization facet, spanning a time frame of 1 to 7 hours. Finally, at the 8-hour mark, a fully covered STO film was evident on both PTO nonpolar facets and positive polarization facet.

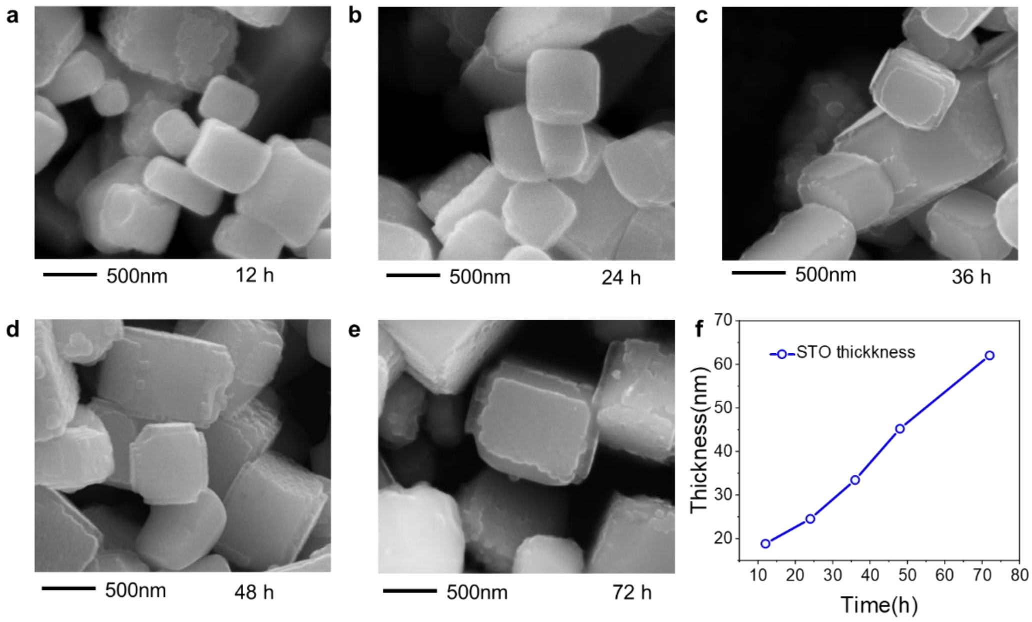

Fig. S19 SEM images of PTO-STO hydrothermally synthesized at different reaction times (second stage: after 8 hours). **a**, 12 hours, **b**, 24 hours, **c**, 36 hours, **d**, 48 hours, **e**, 72 hours. **f**, Time-dependent thickness of STO on PTO surface.

In Fig. S19, the second stage of STO growth on PTO was depicted. Following the formation of a fully covered film, the film served as crystal nucleus. Subsequently, STO continued to grow on the film, resulting in an increase in the thickness of STO with prolonged reaction time. The thickness of STO was observed to increase from approximately 19 nm to 64 nm.

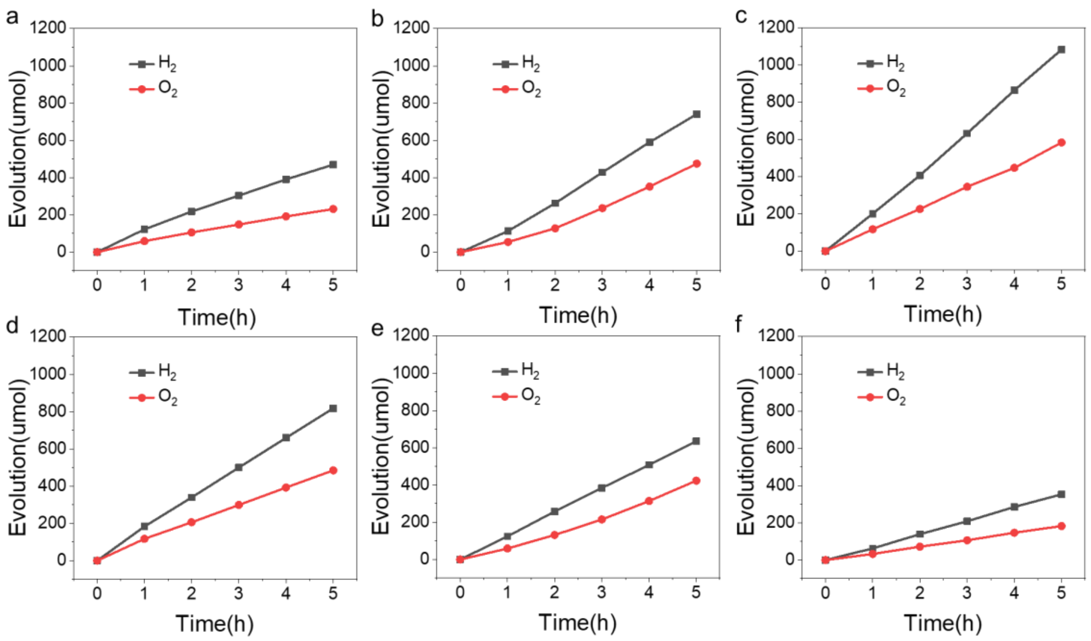

Fig. S20 Overall water splitting reactivity of different PTO-STO samples. **a**, PTO-STO-1, **b**, PTO-STO-4, **c**, PTO-STO-8, **d**, PTO-STO-12, **e**, PTO-STO-24, **f**, PTO-STO-48.

The comprehensive water splitting reactivity of PTO with varying thicknesses of strontium titanate STO is presented in Fig. S20. Notably, PTO-STO-8 exhibits the highest reactivity compared with that of the other samples.

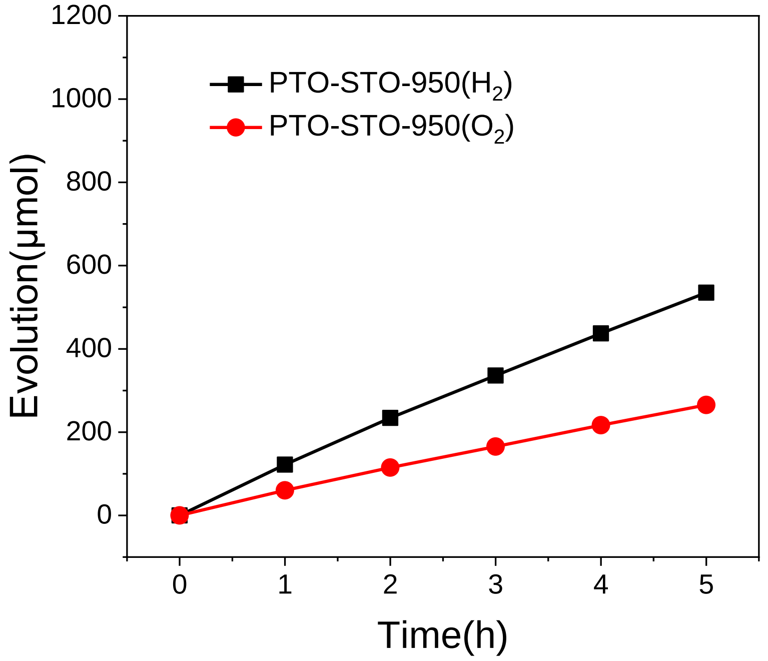

Fig. S21 Overall water splitting reactivity of different PTO-STO samples after annealed at 950 °C

The data on the photocatalytic activity of PTO-STO after heating above its Curie temperature is shown in Fig. S21. After heating PTO to 950 °C, the formation of multidomain structures was observed (as seen in Fig. S2). along with a reduction in the photocatalytic activity of PTO-STO to approximately half of its original level. This decrease in activity is attributed to the change in polarization direction, directly demonstrating the significant impact of ferroelectric properties on photocatalytic performance.

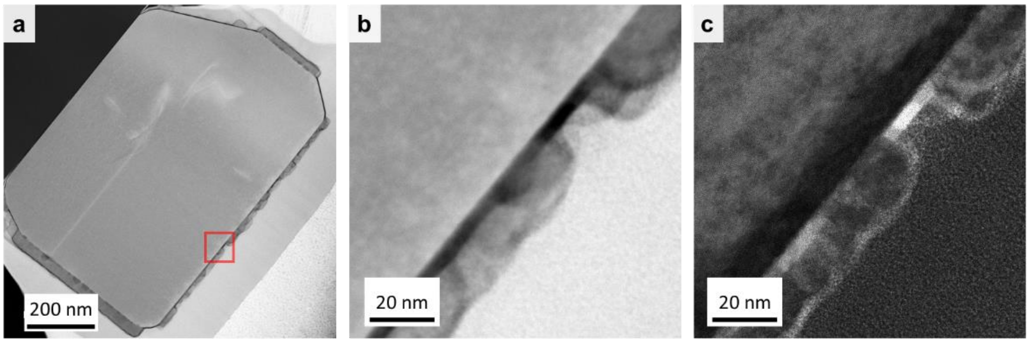

Fig. S22 Surface structure of PTO-STO samples. **a**, the cross-section of PTO-STO. **b**, **c**, the region in (**a**) signed by red rectangle.

Fig. S19 depicts the structure of STO on the facet of PTO after being cut by Ga ion beam. The distribution of STO on the surface of PTO could be categorized into two types. The first type is situated on the nonpolar facet, forming a thicker and complete film. The second type is located on the positive polarization facet, exhibiting a particle morphology. In this instance, the STO film is comprised of individual STO particles, which are thinner than that found on the nonpolar facet.

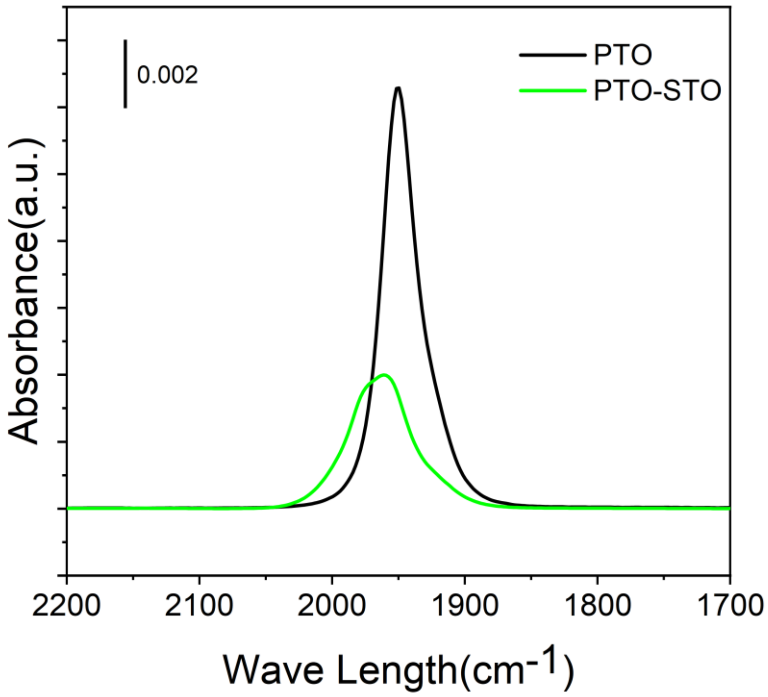

Fig. S23 IR absorption spectrum of CO adsorbed on Rh supported by PTO and PTO-STO samples.

After the growth of STO, a blue shift towards a larger wavenumber was observed in the CO peak. The IR absorption peaks of CO on Rh could be influenced by the nature of the support materials. Rh were photodeposited on the positive polarization facet, where positive bound charge fixed on the surface rendered Rh more electropositive. In contrast, on the STO surface, the absence of the bound charge made Rh less electropositive, resulting in a stronger C=O bond strength than that on PTO surface. Consequently, the CO IR peak exhibited a blue shift. This outcome underscores that Rh was deposited on the STO layer in PTO-STO samples, even when the STO on the positive polarization facet displayed particle morphology.

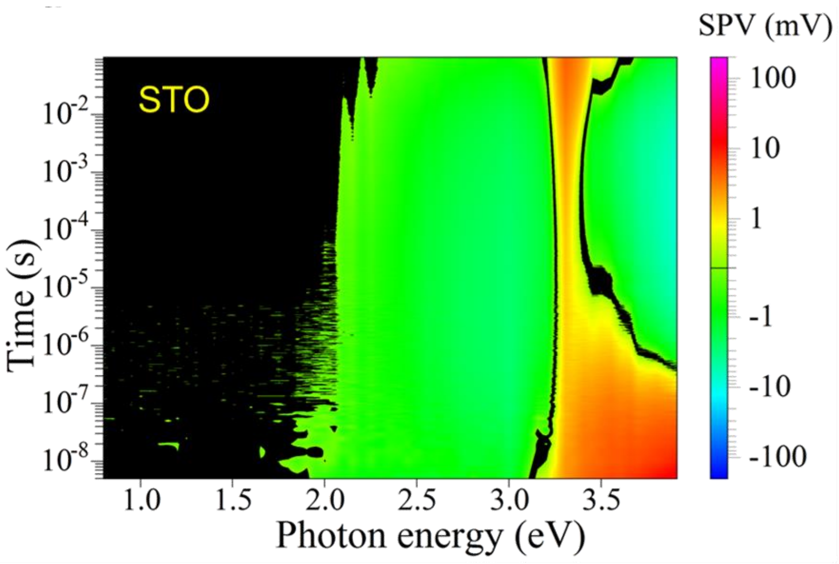

Fig. S24 TPV data of STO.

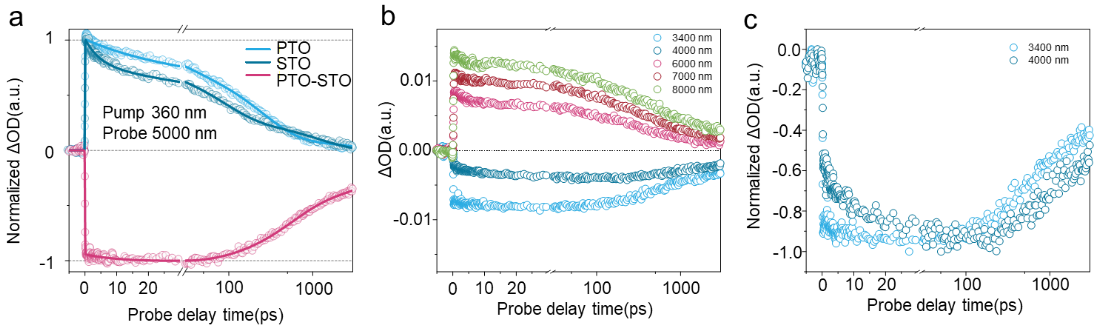

Fig. S25 a, MIR-TAS of PTO, STO, PTO-STO samples. **b,** MIR-TAS of PTO-STO sample with different probe lights. **c,** Normalized MIR-TAS of PTO-STO sample with 3400 and 4000 nm probe light.

In Fig. S25a, both bare PTO and STO exhibited absorption signals in MIR-TAS results, while PTO-STO showed a long-lived bleaching signal. To investigate the formation of the bleaching signal in the PTO-STO sample, the probe light wavelength was varied. As shown in Fig. S25b, the PTO-STO sample exhibited a bleaching signal at probe wavelengths of 3400 nm and 4000 nm. However, when the probe light wavelength was increased to longer than 6000 nm, the signals transitioned to absorption, resembling the shift from ground state bleaching (GSB) to normal absorption observed when the probe wavelength changed from shorter to longer. This indicated that interface states were formed during STO growth. Short-wavelength light could excite these interface states, resulting in a bleaching signal, whereas longer-wavelength light was insufficient to excite these states and thus showed absorption.

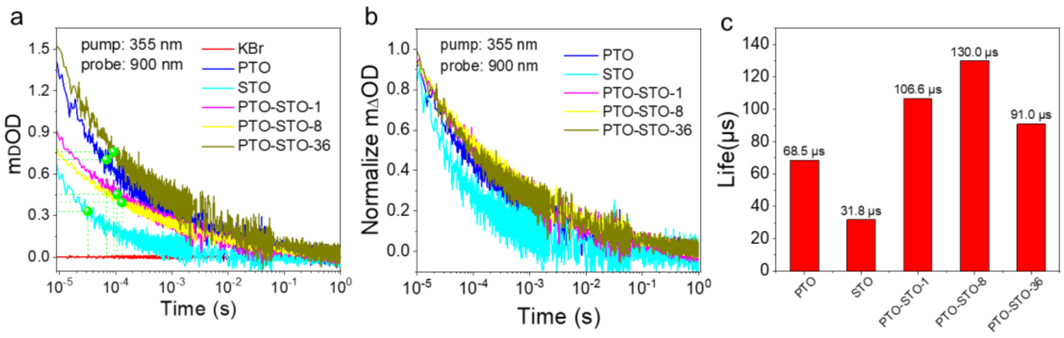

Fig. S26 Slow TAS of different samples. **a**, the decay signal of different samples. Green dots on the line were when the mΔOD decayed to half of the initial. **b**, normalized decay signal of different samples. **c**, Lifetime of different samples obtained from (**a**).

The slow TAS data in Fig. S25 were acquired at 355 nm pump light with energy pulse of 205 μJ and 900 nm probe light. Typically, signal with probe light at this wavelength responded to electrons. After being modified with STO, PTO-STO samples exhibited an extended lifetime of electrons.

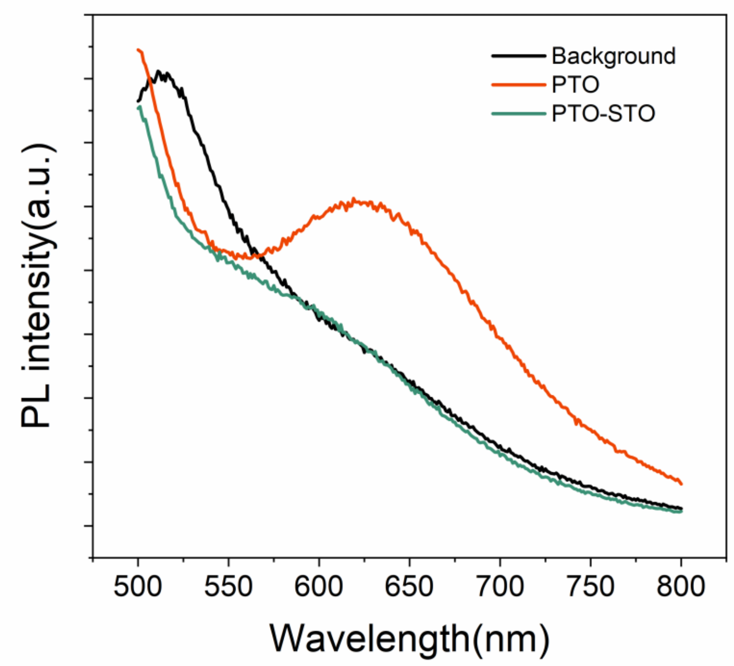

Fig. S27 Photoluminescence (PL) spectrum of PTO before and after STO modification.

PL typically arises from the recombination of electrons and holes in semiconductors. The data showed that PTO-STO samples exhibited a spectrum similar to the background, whereas PTO samples displayed a distinct PL peak around.

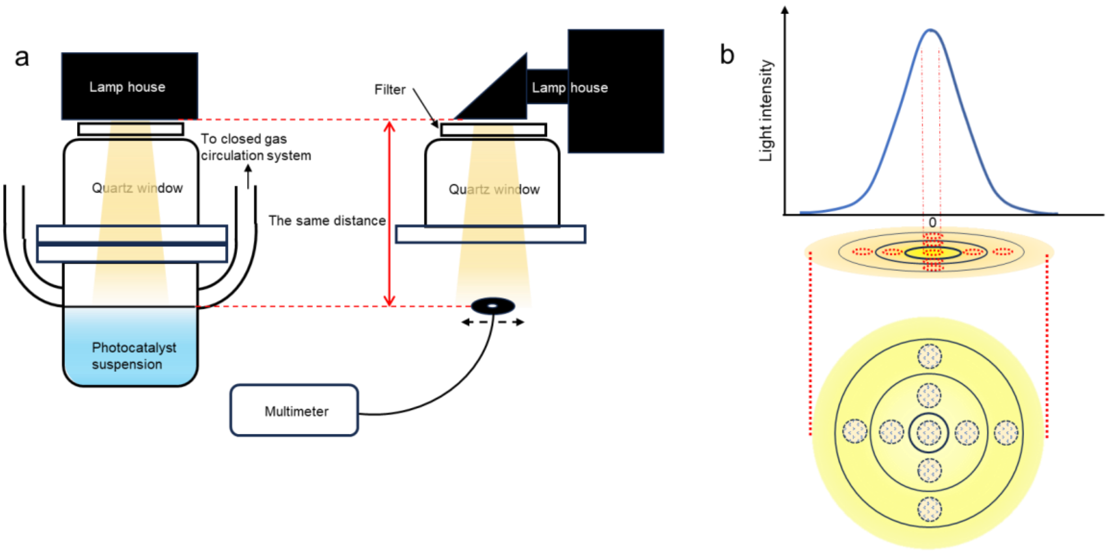

Fig. S28. Schematic of apparent quantum yield (AQY) test. **a**, equipment for the AQY test. **b**, schematic for testing the photon numbers.

The process of calculating the apparent quantum yield (AQY) through photocatalytic hydrogen evolution was based on the equation:

$$AQY = \frac{Number\ of\ reacted\ electrons}{Number\ of\ incident\ photons} \times 100\%$$
$$= \frac{Number\ of\ evolved\ H_2 molecules \times 2}{Photon\ Flux\ \times S \times\ t} \times 100\%$$

Where S represented the irradiation area, and t was the reaction time. The amount of evolved H_2 is tested in the reactor, following the same procedure as overall water splitting with a 365 nm filter between the lamp and the quartz windows. The results are presented in Table S3. The incident light intensity was tested, as shown in Fig. S20, using a method similar to the one applied in Al-doped $SrTiO_3$.[2] In brief, we tested the center of the light and a series of light distributions at different distances from the center. The

number of the photons was calculated by summing the values of photons f(r) at different positions r. The photons numbers at different positions are shown in Fig. S26 and table S2, and the calculation was performed using the following equation:

$$Number\ of\ incident\ photons = \sum_{r=0}^{R} f(r) \times \pi \times (r^2 - (r - \Delta r)^2)$$

Where R was the radius of the reactor.

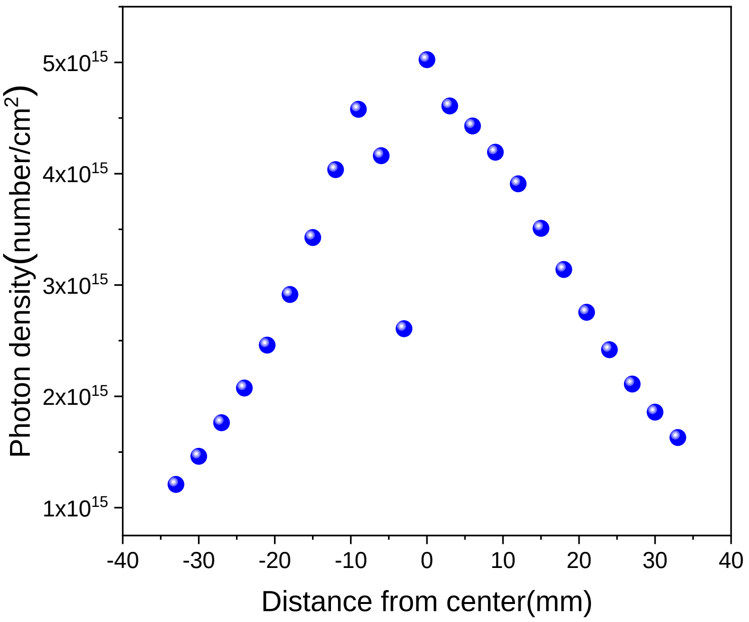

Fig. S29: Photon density at 365 nm in the reactor. The photon density test was conducted at 3 mm intervals. For convenience, the test was carried out without the quartz window. The transmittance of the quartz window was determined to be 0.71513 based on the test results.

Table S1: dynamic fit of MIR-TAS data

	A_1	τ_1(ps)	A_2	τ_2(ps)	A_3	τ_3(ps)
PTO	2.931	22.4±2	3.69	246±13	1.303	3000±800
PTO-STO	0.587	14.8±2.1	-3.155	520±40	-2.75	10000±2600

Three exponents were used for the dynamic fitting of the MIR-TAS of PTO and PTO-STO samples, and the formula was shown as follow:

$$\Delta OD = A_1 e^{-\frac{t}{\tau_1}} + A_2 e^{-\frac{t}{\tau_2}} + A_3 e^{-\frac{t}{\tau_3}} \qquad (1)$$

Table S2: photon distribution in the reactor.

Distance(mm)	Photon density(10^{15}/cm^2)				
	X+	X-	Y+	Y-	average
0	4.85875				4.85875
3	2.60731	4.60814	4.75354	4.68602	4.16375
6	4.16186	4.42853	4.38546	4.32973	4.32639
9	4.57891	4.19309	3.80656	3.88865	4.11680
12	4.03663	3.90944	3.35072	3.40893	3.67643
15	3.42614	3.50905	2.90477	2.91009	3.18751
18	2.91462	3.13962	2.50134	2.38286	2.73461
21	2.46058	2.75571	2.17072		2.46233
24	2.07436	2.410915	1.90470		2.12999
27	1.76331	2.11117	1.61931		1.83126
30	1.46181	1.85828	1.28619		1.53543
33	1.20878	1.62978	0.76544		1.20133

The results were obtained without the quartz window. The transmittance of the quartz window was determined to be 0.71513 based on the test results.

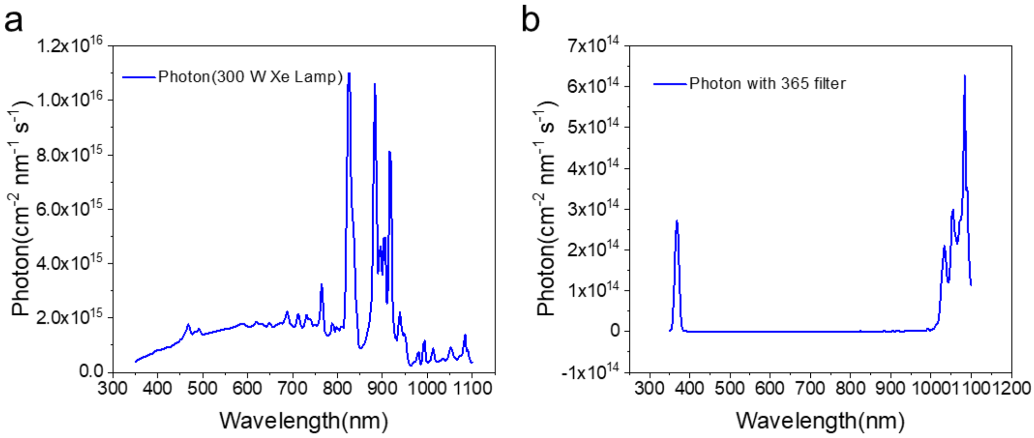

Fig. S30: Photon distribution of Xe lamp. **a,** Photon flux of 300 W Xe lamp under photocatalysis. **b**, Photon flux of 300 W Xe lamp with 365 nm band-pass filter.

It was worth noting that the photon flux in Fig. S20 was different from that in Fig. S27. From Fig S28b, we could see that, the passed light through 365 nm band-pass filter was distributed from around 340 to 390 nm. During AQY test (Fig S19), the photon flux was the sum of all the photon between 340 to 390 nm, so Fig. S27 covered more wavelength and showed more photon flux compared with that in Fig. S28.

Table S3: photocatalysis water splitting at 365 nm.

Time(h)	H_2 Evolution(μmol)	O_2 Evolution(μmol)
0	0	0
0.5	4.4037	3.3114
1	8.7625	7.3510
1.5	12.3328	8.6285
2	15.3391	9.8142

From the data in Table S2, the number of photons at 365 nm was 373.60 μmol/h, and according to the data in Table S3, the average H_2 evolution was 7.67 μmol/h. Therefore, the AQY was approximately 4.08% at 365 nm, which stands out as the highest among ferroelectric photocatalysts.

Table S4: AQY results of ferroelect ric photocatalyst.

Photocatalysts	H_2 evolution (μmol/h)	O_2 evolution (μmol/h)	AQY(%)	Ref.
$PbTiO_3$-$SrTiO_3$	216.83	116.90	4.08 (365 nm)	This work
Bi_3TiNbO_9-W	106.71	47.94	0.57 (365 nm)	[3]
Bi_3TiNbO_9	21.78	9.94	0.26 (365 nm)	[4]
$PbTiO_3$	3.29	1.74	0.071 (365 nm)	[5]
$PbTiO_3$/$BiVO_4$	48.04	24.19	-	[6]
Bi_3TiNbO_9	17.13	13.76	-	[7]
$Bi_3Ti_{0.8}Cr_{0.1}Nb_{1.1}O_9$	41.11		0.52 (⩾250 nm)	[8]
Bi_3TiNbO_9/rGO	2.4		-	[9]

Table S5: dynamic fit of TPV data

	PTO	PTO-STO
A_1	/	84 mV
β_1	/	0.89
$\boldsymbol{\tau}_1$	/	340 μs
A_2	-121 mV	-110 mV
β_2	0.2	0.36
τ_2	50 μs	9 ms
B	-2e-4 mV	-3e-5 mV
α	0.32	0.33

It shall be possible to fit the transients with a minimum of time dependent functions, one for each dominating process. SPV transients can often be fitted with stretched exponentials (equation (2)) or by power law dependencies (equation (3)) depending on whether a process is limited more, for example, by a Gaussian-like distribution of defects or by an exponential distribution of defects or dispersive transport, respectively.

$$SPV(t) = A \cdot exp\left(-\left(\frac{t-t_0}{\tau}\right)^{\beta}\right) \qquad (2)$$

$$SPV(t) = B \cdot \left(\frac{1}{t-t_0}\right)^{\alpha} \qquad (3)$$

Whereas A, β and τ and B and α are amplitude, stretching parameter and time constant describing a stretched exponential and the amplitude and

power coefficient describing a power law, respectively. The value of t_0 is related to the onset of the laser pulse.

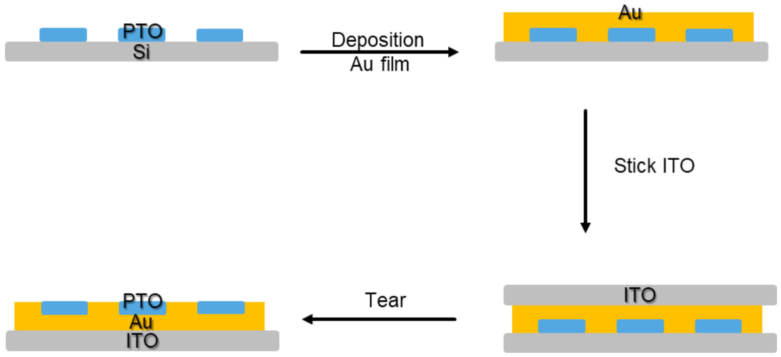

Fig. S31: Schematic of PFM Sample Preparation Procedure.

The preparation of PFM samples was carried out using the following method: First, the particles were dispersed on a monocrystal polished Si surface. Next, a layer of Au film was evaporated onto the Si surface. Subsequently, the Au film was adhered to a conductive ITO surface using an adhesive layer. Finally, the Si substrate was peeled away, transferring the particles and Au film onto the ITO substrate. Before testing, electrical contact was established by connecting the Au film and exposed ITO with conductive Ag adhesive.

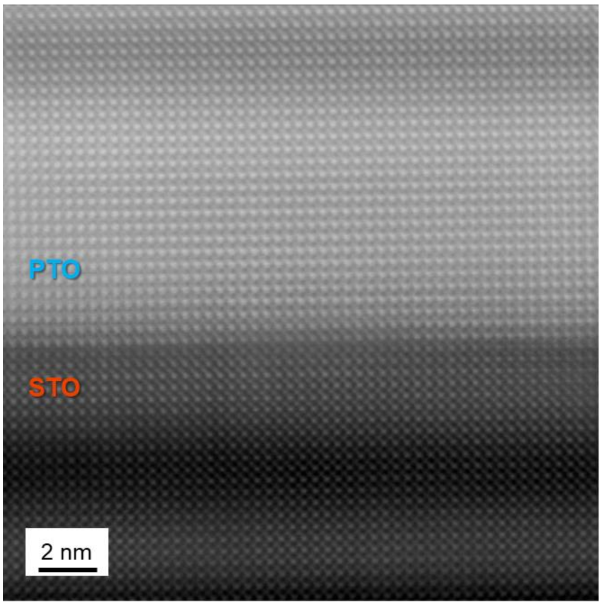

Fig. S32: repeat results of PTO-STO interface.

The PTO-STO interface was examined using additional particles. As shown in Fig. S32, a well-structured interface was observed, consistent with the results in Fig. 3c, further verifying the reliability of our findings that, after modification with STO nanolayers, surface defects in PTO were effectively eliminated.

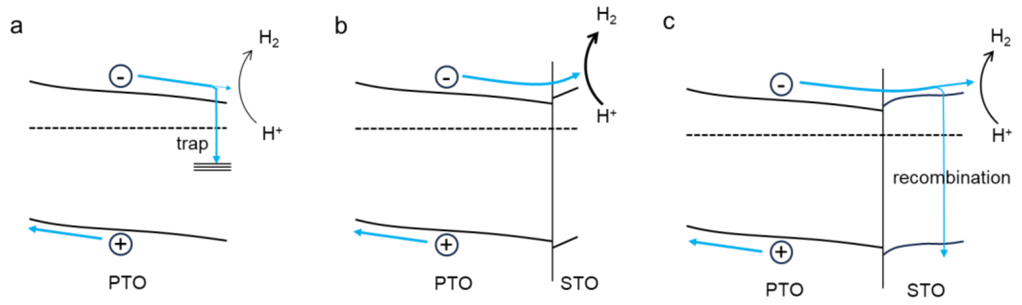

Fig. S33: Schematic of charge separation in various samples. **a,** Charge separation in bare PTO. **b,** Charge separation in PTO-STO samples with a thinner modification layer. **c,** Charge separation in PTO-STO samples with a thicker modification layer. The blue arrows represent the charges migrate directions.

Fig. S33 illustrated charge separation across different samples. In bare PTO, as shown in Fig. S33a, electrons separated toward the positive polarization facet. However, when they reached the surface, significant surface recombination occurred, limiting the photocatalytic activity. After the growth of STO nanolayers, as shown in Fig. S33b, the elimination of surface defects allowed electrons to transfer more readily to the STO surface and participate in H_2 production. However, when the STO layer was much thicker, the extended travel distance for electrons led to increased charge recombination within the STO layer, decreasing the number of electrons available for water splitting.

Reference

1 Du, P. *et al.* Reconstructing Ferroelectric Polarization Screening of $PbTiO_3$ by Epitaxial $SrTiO_3$ for Efficient Photocatalytic Overall Water Splitting. *Advanced Functional Materials* **n/a**, 2312888, doi:10.1002/adfm.202312888 (2024).

2 Takata, T. *et al.* Photocatalytic water splitting with a quantum efficiency of almost unity. *Nature* **581**, 411-414, doi:10.1038/s41586-020-2278-9 (2020).

3 Huang, J. *et al.* Gradient tungsten-doped Bi_3TiNbO_9 ferroelectric photocatalysts with additional built-in electric field for efficient overall water splitting. *Nat Commun* **14**, 7948, doi:10.1038/s41467-023-43837-4 (2023).

4 Huang, J. *et al.* Selective Exposure of Robust Perovskite Layer of Aurivillius-Type Compounds for Stable Photocatalytic Overall Water Splitting. *Adv Sci (Weinh)* **10**, e2302206, doi:10.1002/advs.202302206 (2023).

5 Wan, G. *et al.* Photocatalytic Overall Water Splitting over $PbTiO_3$ Modulated by Oxygen Vacancy and Ferroelectric Polarization. *J Am Chem Soc* **144**, 20342-20350, doi:10.1021/jacs.2c08177 (2022).

6 Kang, Y. *et al.* Ferroelectric polarization enabled spatially selective adsorption of redox mediators to promote Z-scheme photocatalytic overall water splitting. *Joule* **6**, 1876-1886, doi:10.1016/j.joule.2022.06.017 (2022).

7 Yin, X. *et al.* Realizing selective water splitting hydrogen/oxygen evolution on ferroelectric Bi_3TiNbO_9 nanosheets. *Nano Energy* **49**, 489-497, doi:10.1016/j.nanoen.2018.05.001 (2018).

8 Jiang, L., Ni, S., Liu, G. & Xu, X. Photocatalytic hydrogen production over Aurivillius compound Bi_3TiNbO_9 and its modifications by Cr/Nb co-doping. *Applied Catalysis B: Environmental* **217**, 342-352, doi:10.1016/j.apcatb.2017.06.012 (2017).

9 Bai, J., Chen, C., Zheng, J. & Guo, C. Regulation of ferroelectric polarization and reduced graphene oxide (RGO) synergistically promoting photocatalytic performance of Bi_3TiNbO_9. *Materials Today Physics* **24**, doi:10.1016/j.mtphys.2022.100691 (2022).
